# Depression and anxiety of medical students at Kunming Medical University during COVID-19: A cross-sectional survey

**DOI:** 10.3389/fpubh.2022.957597

**Published:** 2022-09-07

**Authors:** Ying Guo, Shunda Li, Lanchun Zhang, Qun Xuan, Liu He, Qingyan Ye, Jiaqing Ma, Li Peng, Yunxia Xiong, Jianyu Yang, Haofei Yu, Jianping Xie, Heng Shao, Yun Yuan

**Affiliations:** ^1^Faculty of Basic Medical Sciences, Kunming Medical University, Kunming, China; ^2^School of Pharmaceutical Science, Department of Zoology and Yunnan Key Laboratory of Pharmacology for Natural Products, Kunming Medical University, Kunming, China; ^3^Library, Yunnan Minzu University, Kunming, China; ^4^Department of Geriatrics, First People's Hospital of Yunnan Province and Affiliated Hospital of Kunming University of Science and Technology, Kunming, China

**Keywords:** depression, anxiety, COVID-19, medical students, mental health

## Abstract

An isolation strategy was used to control the transmission and rapid spread of COVID-19 in Yunnan. As a result, students were supposed to stay at home and disrupted their outside activities. It led to a detrimental influence on students' mental health. The purpose of this study was to investigate the prevalence and risk factors of depression and anxiety among medical students and to provide ideas for the prevention of depression and anxiety in medical students. A cross-sectional survey was conducted among 2,116 medical students at Kunming Medical University from July 8 to July 16, 2020. Participants' demographic and living conditions were collected. Depression and anxiety were measured using the Patient Health Questionnaire 9 and General Anxiety Disorder-7, respectively. Uni- and multivariate logistic regression analyses were performed to detect risk factors associated with depression and anxiety. The prevalence rates of depression and anxiety among medical students were 52.5 and 29.6%, respectively. Depression was more likely to be caused by low grades, lack of physical exercise, drug use, irregular diet, extensive screen time on mobile phones, being greatly affected by the COVID-19 pandemic, and inadaptability to offline courses. Anxiety was more likely to be caused by lack of physical exercise, drug use, irregular diet, and inadaptability to offline courses. Depression and anxiety are highly comorbid. Our study showed predictive factors for depression and anxiety and identified a major mental health burden on medical students during the COVID-19 outbreak. More targeted measures should be taken to improve the mental state of students to reduce the incidence of depression and anxiety.

## Highlights

- Depression and anxiety were associated with living habits (lack of physical exercise and drug use).
- Depression and anxiety were related to irregular diet.
- Depression showed the strongest association with extensive screen time on mobile phones.
- Depression was associated with the influence of the COVID-19 pandemic.
- Depression and anxiety were related to the inadaptability of offline courses.

## Introduction

COVID-19 infection spread rapidly and frequently starting at the end of 2019, and the worldwide pandemic has become the most serious global public health problem currently (1). The epidemic of COVID-19 has widely affected the lifestyle of global individuals after the suppression strategy at the beginning of 2020, including the shutdown of public places, closing of public transport, and travel restrictions, to prevent the transmission of the virus from person to person (2). After the outbreak of the COVID-19 epidemic occurred, the Chinese government took strong management measures such as home quarantine to control the crisis. The teaching model of the colleges and universities in Yunnan province changed from teaching in the classroom to teaching online in March 2020, and online services helped students continue their educational courses in the advent of the current pandemic COVID-19 (3, 4). While these measures effectively alleviated the risk of epidemic spread among college students in Yunnan province, University students were at particularly high risk for mental disorders, such as major depressive disorder, anxiety, and psychiatric comorbidities (5). Depression and anxiety are considered the most common mental health disorders. Depressive disorders and anxiety may produce negative outcomes, such as underachievement, reduced quality of life, and an increased risk of suicide (6). Medical students experience a heavy workload, long hours of clinical training, and a lack of rest. These pressures greatly impact the mental health of medical students (7). Because of the ongoing impact on University students and their families amid the COVID-19 crisis, including job stress and job turnover intention (8), anxiety and depressive disorders cause a higher societal burden than any other mental health disorder (9).

The COVID-19 pandemic has not only affected the teaching model of the University, but also the way students live in. With the increasing frequency of social media use in home quarantine (10, 11), space restrictions on outdoor activity and the need to stay home have resulted in reduced physical exercise and increased sedentary behavior (12). Moreover, diet consumption and patterns are more unhealthy during home confinement (13). The University switched the teaching model from teaching online to teaching offline in June 2020. Lifestyle changes and maladjustment to offline courses may affect the mental health of medical students.

**Figure 1:**
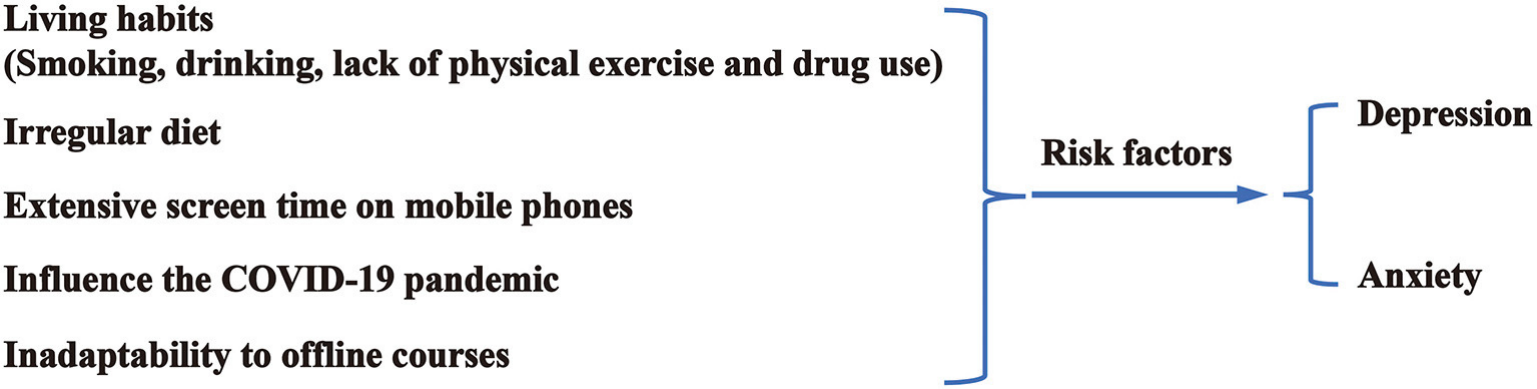
Proposed research framework.

A detailed understanding of the depression and anxiety status of medical students can help medical students to receive treatment as soon as possible. However, the mental status of students at Kunming Medical University is still poorly understood, and factors associated with increased risks for depression and anxiety remain unclear. Based on these considerations, the present study aimed to explore the prevalence of depression, anxiety, living habits (smoking, drinking, exercising, and taking medication), irregular diet, time spent on the smartphone, the influence of COVID-19, and inadaptability to offline teaching, determine the potential predictive factors for depression and anxiety (Figure 1), and provide advice about proper intervention management to help medical students maintain their mental fitness during the COVID-19 epidemic.

## Materials and methods

### Study design

This cross-sectional survey aimed to investigate the subjects' depressive symptoms and anxiety. Through the head teacher of each class, we distributed an invitation to undergraduates in clinical medicine to complete a web-based questionnaire. All invited students could see this questionnaire. All replies were anonymous, and participation was completely voluntary. The survey was conducted at Kunming Medical University from July 8 to July 16, 2020. A stratified sampling method was adopted to recruit undergraduates in clinical medicine from students in the first (freshmen), second (sophomores), and third grades (juniors). We used a networking questionnaire tool (Questionnaire Star Software) to transfer the paper-based questionnaire to an online questionnaire. After we checked whether the online questionnaire was consistent with the paper version, ten students were invited to fill out this questionnaire to test whether it was comprehensible. The questionnaire included four parts: basic information, living conditions, assessment of the depressive state, and assessment of anxiety. Basic information included age, gender, grade, native place (hometown), and if they were the only child in their family. Living conditions were evaluated by eight items: smoking, drinking, exercising, taking medicine, diet, time spent on the smartphone, the impact of COVID-19 on one's life, and the degree of adaptation to the teaching methods of courses.

### Sampling and sample size

A simple random sampling method was used to estimate the required sample size. Prevalence for depression and anxiety was set as 32.74 and 27.22% (14). The acceptable error rate was 8%, and the effective response rate was 80%. Therefore, the adjusted required sample size was 190.

### Data collection

Data were collected through online questionnaires. Among the 2,116 medical students from Kunming Medical University who completed the online questionnaire, 68 participants were excluded due to non-standard fillings (the completion time was <2 min or when the questionnaire was incomplete). The final number of study samples was 2,048.

### Ethical considerations

The research at Kunming Medical University was implemented after the study protocol has been approved by the Research Ethics Committees of Kunming Medical University.

### Measurement

#### General characteristics

In this section, questions were asked regarding demographics, such as age, gender, grade, native place (hometown), and if they were the only child in their family. Living conditions were evaluated by five aspects: living habits (smoking, drinking, exercising, taking medicine), diet, time spent on the smartphone, the impact of COVID-19 on one's life, and the degree of adaptation to the teaching methods of courses.

##### Depression

The Chinese version of the Patient Health Questionnaire 9 (PHQ-9) (15) was used to evaluate the outcomes of depressive symptoms over the previous month. The scale comprised nine questions rated on a four-point scale (0–3). The total scores of 0–4, 5–9, 10–14, 15–19, and 20–27 were categorized as minimal, mild, moderate, moderately severe, and severe depressive symptoms, respectively. A total score >5 was adopted in the research. The Cronbach's α for PHQ-9 was 0.898 (Bootstrap 95% CI: 0.891–0.904) in this sample.

##### Anxiety

The Chinese version of the General Anxiety Disorder-7 (GAD-7) (16) was applied to assess the students' anxiety over the previous month. The GAD-7 is a brief, self-reported scale composed of seven items with a 4-point Likert scale ranging from 0–3. Total scores of 0–4, 5–9, 10–14, and 15–21 were defined as minimal, mild, moderate, and severe symptoms of anxiety, respectively. A total score >5 was used in this study. The Cronbach's α for GAD-7 was 0.934 (Bootstrap 95% CI: 0.929–0.938) in this sample.

### Statistical analysis

All data were analyzed by IBM SPSS Statistics version 26.0. Categorical variables are described as numbers and proportions (%), and the chi-square test or Fisher's test was used to compare the proportions of depression or anxiety, if applicable. We used binary logistic regression to examine the risk factors for depression and anxiety. Only variables with a *P*-value under 0.05 were included in multivariate regression analysis. The associations between dependent variables and potential risk factors were examined through multivariate logistic regression analysis. The results of the regression analysis are presented with odds ratios (ORs) and 95% confidence intervals (CIs). A *P*-value <0.05 was considered to be statistically significant.

## Results

### Characteristics of participants

The final study sample was 2,048 participants with an average age of 20.35 years (SD: 1.304, range, 17–24) and 40.1% male. Freshmen, sophomores, and juniors made up 30.1, 37.6, and 32.3% of the total sample, respectively. Students from Yunnan Province accounted for 95.3% of the total. Those who identified as only children accounted for 23.7%. Of the participants, 52.5% were depressive, and 29.6% were anxious (Tables 1, 2).

**Table 1:** Characteristics of enrolled students divided into non-depression and depression groups (*N* = 2,048).

**Variables**	**Total (*N =* 2,048)**	**Non-depression (*N =* 972)**	**Depression (*N =* 1,076)**	**χ^2^**	***P*-value**
	***n* (%)**	***n* (%)**	***n* (%)**		
**Gender**				0	0.991
Male	822 (40.1)	390 (47.4)	432 (52.6)		
Female	1,226 (59.9)	582 (47.5)	644 (52.5)		
**Grade**				5.977	0.05
Freshmen	616 (30.1)	282 (45.8)	334 (54.2)		
Sophomores	770 (37.6)	350 (45.5)	420 (54.5)		
Juniors	662 (32.3)	340 (51.4)	322 (48.6)		
**Native place**				3.904	0.048
Yunnan Province	1,952 (95.3)	917 (47.0)	1,035 (53.0)		
Outside Yunnan Province	96 (4.7)	55 (57.3)	41 (42.7)		
**Only child**				0.251	0.616
Yes	485 (23.7)	235 (48.5)	250 (51.5)		
No	1,563 (76.3)	737 (47.2)	826 (52.8)		
**Smoking**				5.175	0.159
None	1,943 (94.9)	933 (48.0)	1,010 (52.0)		
Once per week	25 (1.2)	10 (40.0)	15 (60.0)		
Twice per week	19 (0.9)	8 (42.1)	11 (57.9)		
More than twice per week	61 (3.0)	21 (34.4)	40 (65.6)		
**Drinking**					<0.001
None	1,913 (93.4)	929 (48.6)	984 (51.4)		
Once per week	125 (6.1)	41 (32.8)	84 (67.2)		
Twice per week	5 (0.2)	0 (0)	5 (100)		
More than twice per week	5 (0.2)	2 (40.0)	3 (60.0)		
**Exercising**				17.686	0.001
None	171 (8.3)	76 (44.4)	95 (55.6)		
Once per week	387 (18.9)	149 (38.5)	238 (61.5)		
Twice per week	783 (38.2)	387 (49.4)	396 (50.6)		
More than twice per week	707 (34.5)	360 (50.9)	347 (49.1)		
**Taking medicine**				54.787	<0.001
None	1,450 (70.8)	762 (52.6)	688 (47.4)		
Once per week	395 (19.3)	149 (37.7)	246 (62.3)		
Twice per week	112 (5.5)	34 (30.4)	78 (69.6)		
More than twice per week	91 (4.4)	27 (29.7)	64 (70.3)		
**Irregular diet**				173.59	<0.001
None	1,039 (50.7)	636 (61.2)	403 (38.8)		
Once per week	465 (22.7)	184 (39.6)	281 (60.4)		
Twice per week	359 (17.5)	99 (27.6)	260 (72.4)		
More than twice per week	185 (9.0)	53 (28.6)	132 (71.4)		
**Time spent on the smartphone**				64.624	<0.001
<30 min per day	57 (2.8)	42 (73.7)	15 (26.3)		
30–60 min per day	268 (13.1)	150 (56.0)	118 (44.0)		
1–2 h per day	593 (29.0)	329 (55.5)	264 (44.5)		
More than 2 h per day	1,130 (55.2)	451 (39.9)	679 (60.1)		
**Influence of COVID-19**				84.69	<0.001
None	223 (10.9)	157 (70.4)	66 (29.6)		
Small influence	1,185 (57.9)	584 (49.3)	601 (50.7)		
Moderate influence	502 (24.5)	190 (37.8)	312 (62.2)		
Great impact	138 (6.7)	41 (29.7)	97 (70.3)		
**Offline teaching**				76.085	<0.001
Well-adapted	291 (14.2)	183 (62.9)	108 (37.1)		
More adaptable	1,480 (72.3)	715 (48.3)	765 (51.7)		
Almost inadaptable	246 (12.0)	65 (26.4)	181 (73.6)		
Inadaptable	31 (1.5)	9 (29.0)	22 (71.0)		
**Anxiety**				653.143	<0.001
No	1,442 (70.4)	948 (65.7)	494 (34.3)		
Yes	606 (29.6)	24 (4.0)	582 (96.0)		

**Table 2:** Characteristics of enrolled students divided into non-anxiety and anxiety groups (*N* = 2,048).

**Variables**	**Total (*N =* 2,048)**	**Non-anxiety (*N =* 1,442)**	**Anxiety (*N =* 606)**	**χ^2^**	***P*-value**
	***n* (%)**	***n* (%)**	***n* (%)**		
**Gender**				3.081	0.158
Male	822 (40.1)	561 (68.2)	261 (31.8)		
Female	1,226 (59.9)	881 (71.9)	345 (28.1)		
**Grade**				3.685	0.435
Freshmen	616 (30.1)	416 (67.5)	200 (32.5)		
Sophomores	770 (37.6)	548 (71.2)	222 (28.8)		
Juniors	662 (32.3)	478 (72.2)	184 (27.8)		
**Native place**				0.609	0.279
Yunnan Province	1,952 (95.3)	1,371 (70.2)	581 (29.8)		
Outside Yunnan Province	96 (4.7)	71 (74.0)	25 (26.0)		
**Only child**				1.173	0.561
Yes	1,563 (76.3)	351 (72.4)	134 (27.6)		
No	484 (23.7)	1,091 (69.8)	472 (30.2)		
**Smoking**				2.055	0.561
None	1,943 (94.9)	1,370 (70.7)	570 (29.3)		
Once per week	25 (1.2)	18 (72.0)	7 (28.0)		
Twice per week	19 (0.9)	13 (68.4)	6 (31.6)		
More than twice per week	61 (3.0)	38 (62.3)	23 (37.7)		
**Drinking**					0.022
None	1,913 (93.4)	1,361 (71.1)	552 (28.9)		
Once per week	125 (6.1)	76 (60.8)	49 (39.2)		
Twice per week	5 (0.2)	2 (40.0)	3 (60.0)		
More than twice per week	5 (0.2)	3 (60.0)	2 (40.0)		
**Exercising**				17.622	0.001
None	171 (8.3)	100 (58.5)	71 (41.5)		
Once per week	387 (18.9)	264 (68.2)	123 (31.8)		
Twice per week	783 (38.2)	580 (74.1)	203 (25.9)		
More than twice per week	707 (34.5)	498 (70.4)	209 (29.6)		
**Taking medication**				51.462	<0.001
None	1,450 (70.8)	1,075 (74.1)	375(25.9)		
Once per week	395 (19.3)	264 (66.8)	131 (33.2)		
Twice per week	112 (5.5)	61 (54.5)	51 (45.5)		
More than twice per week	9 (4.4)	42 (46.2)	49 (53.8)		
**Irregular diet**				105.854	<0.001
None	1,039 (50.7)	827 (79.6)	212 (20.4)		
Once per week	465 (22.7)	313 (67.3)	152 (32.7)		
Twice per week	359 (17.5)	209 (58.2)	150 (41.8)		
More than twice per week	185 (9.0)	93 (50.3)	92 (49.7)		
**Time spent on the smartphone**				35.093	<0.001
<30 min per day	57 (2.8)	45 (78.9)	12 (2.1)		
30–60 min per day	268 (13.1)	215 (80.2)	53 (19.8)		
1–2 h per day	593 (29.0)	445 (75.0)	148 (25.0)		
More than 2 h per day	1,130 (55.2)	737 (65.2)	393 (34.8)		
**Influence of COVID-19**				65.413	<0.001
None	223 (10.9)	190 (85.2)	33 (14.8)		
Small influence	1,185 (57.9)	867 (73.2)	318 (26.8)		
Moderate influence	502 (24.5)	313 (62.4)	189 (37.6)		
Great impact	138 (6.7)	72 (52.2)	66 (47.8)		
**Offline teaching**				80.704	<0.001
Well-adapted	291 (14.2)	239 (82.1)	52 (17.9)		
More adaptable	1,480 (72.3)	1,065 (72.0)	415 (28.0)		
Almost inadaptable	246 (12.0)	127 (51.6)	119 (48.4)		
Inadaptable	31 (1.5)	11 (35.5)	20 (64.5)		
**Depression**				653.143	<0.001
No	972 (47.5)	948 (97.5)	24 (2.5)		
Yes	1,076 (52.5)	494 (45.9)	582 (54.1)		

### Prevalence of depression

Detailed demographics and living conditions of the total sample and the 2 subpopulations related to depression are shown in Table 1. The prevalence of depression showed that more than half of the students (52.5%) suffered from depression. The variables native place, drinking, exercising, taking medicine, irregular diet, surfing the internet, the influence of COVID-19, offline teaching, and anxiety were significantly related to the prevalence of depression (*P* < 0.05, Table 1).

### Prevalence of anxiety

A total of 29.6% of the students were subjected to anxiety. The variables drinking, exercising, taking medicine, irregular diet, surfing the internet, the influence of COVID-19, offline teaching, and depression were significantly different (*P* < 0.05, Table 2).

### Risk factors for depression or anxiety

All variables related to depression or anxiety were analyzed in univariate and multivariate logistic regression. The factors with a *P*-value < 0.05 in the univariate analysis were included in the final multivariate analysis to adjust for potential confounding factors.

The results (Table 3) indicated that freshmen [odds ratio (OR) = 1.376, 95%CI: 1.028–1.841; *P* = 0.032], sophomores (OR = 1.544, 95% CI: 1.175–2.028; *P* = 0.002), exercising once per week (OR = 1.945, 95% CI: 1.411–2.682; *P* < 0.001), taking medicine once per week (OR = 1.511, 95% CI: 1.135–2.011; *P* = 0.005), irregular diet once per week (OR = 2.006, 95 CI%: 1.516–2.655; *P* < 0.001), irregular diet twice per week (OR = 2.862, 95% CI: 2.077–3.943; *P* < 0.001), irregular diet more than twice per week (OR = 1.912, 95% CI: 1.234–2.962; *P* = 0.004), spending more than 2 h per day on the smartphone (OR = 3.087, 95 CI%: 1.276–7.470; *P* = 0.012), small impact from COVID−19 (OR = 1.852, 95% CI: 1.242–2.762; *P* = 0.003), moderate impact from COVID−19 (OR = 2.389, 95% CI: 1.532–3.726; *P* < 0.001), great impact from COVID−19 (OR = 3.046, 95% CI: 1.657–5.601; *P* < 0.001), considering offline teaching almost inadaptable (OR = 2.376, 95% CI: 1.473–3.833; *P* < 0.001), and anxiety (OR = 41.693, 95% CI: 26.915–64.586; *P* < 0.001) were associated with a higher risk of depression.

**Table 3:** Logistic regression analysis for risk factors of depression.

**Variables**	**Univariate**		**Multivariate**	
	**OR (95% CI)**	***P*-value**	**OR (95% CI)**	***P*-value**
**Gender**				
Male	1			
Female	1.195 (0.927–1.542)	0.169		
**Grade**				
Freshmen	1.287 (0.900–1.842)	0.167	1.376 (1.028–1.841)	0.032
Sophomores	1.459 (1.082–1.969)	0.013	1.544 (1.175–2.028)	0.002
Juniors	1		1	
**Native place**				
Yunnan Province	1			
Outside Yunnan Province	0.674 (0.384–1.183)	0.169		
**Only child**				
Yes	1.007 (0.767–1.323)	0.957		
No	1			
**Smoking**				
None	1			
Once per week	1.661 (0.603–4.575)	0.326		
Twice per week	0.959 (0.284–3.234)	0.946		
More than twice per week	1.559 (0.760–3.195)	0.225		
**Drinking**				
None	1			
Once per week	1.466 (0.871–2.468)	0.150		
More than or equal to twice per week	2.623 (0.403–17.077)	0.313		
**Exercising**				
None	1.136 (0.692–1.864)	0.614	1.095 (0.671–1.788)	0.715
Once per week	1.982 (1.428–2.750)	<0.001	1.945 (1.411–2.682)	<0.001
Twice per week	1.202 (0.918–1.575)	0.181	1.203 (0.924–1.566)	0.171
More than twice per week	1		1	
**Taking medication**				
None	1		1	
Once per week	1.508 (1.128–2.018)	0.006	1.511 (1.135–2.011)	0.005
Twice per week	1.477 (0.865–2.524)	0.153	1.547 (0.909–2.634)	0.108
More than twice per week	1.211 (0.654–2.242)	0.543	1.306 (0.707–2.410)	0.394
**Irregular diet**				
None	1		1	
Once per week	1.971 (1.487–2.612)	<0.001	2.006 (1.516–2.655)	<0.001
Twice per week	2.774 (2.006–3.836)	<0.001	2.862 (2.077–3.943)	<0.001
More than twice per week	1.869 (1.201–2.908)	0.006	1.912 (1.234–2.962)	0.004
**Time spent on the smartphone**				
<30 min per day	1		1	
30–60 min per day	2.058 (0.810–5.229)	0.129	2.160 (0.859–5.432)	0.101
1–2 h per day	1.789 (0.722–4.428)	0.209	1.876 (0.766–4.596)	0.169
More than 2 h per day	2.920 (1.193–7.149)	0.019	3.087 (1.276–7.470)	0.012
**Influence of COVID-19**				
None	1		1	
Small influence	1.824 (1.221–2.727)	0.003	1.852 (1.242–2.762)	0.003
Moderate influence	2.349 (1.502–3.675)	<0.001	2.389 (1.532–3.726)	<0.001
Great impact	3.038 (1.644–5.615)	<0.001	3.046 (1.657–5.601)	<0.001
**Offline teaching**				
Well-adapted	1		1	
More adaptable	1.356 (0.965–1.904)	0.079	1.360 (0.971–1.905)	0.073
Almost inadaptable	2.378 (1.464–3.865)	<0.001	2.376 (1.473–3.833)	<0.001
Inadaptable	0.917 (0.262–3.214)	0.892	0.946 (0.278–3.223)	0.930
**Anxiety**				
No	1		1	
Yes	42.598 (27.433–66.145)	<0.001	41.693 (26.915–64.586)	<0.001

The results (Table 4) showed that taking medicine more than twice per week (OR = 2.310, 95% CI: 1.322–4.036; *P* = 0.003), irregular diet more than twice per week (OR = 1.843, 95% CI: 1.225–2.773; *P* = 0.003), considering offline teaching almost inadaptable (OR = 2.225, 95% CI: 1.385–3.575; *P* = 0.001), considering offline teaching inadaptable (OR = 6.018, 95% CI: 1.990–18.203; *P* = 0.001), and depression (OR = 42.480, 95% CI: 27.560–65.476; *P* < 0.001) were related to a higher risk for anxiety. Exercising once per week (OR = 0.720, 95% CI: 0.519–0.999; *P* = 0.049) and twice per week (OR = 0.706, 95% CI: 0.532–0.938; *P* = 0.016) showed a lower risk for anxiety.

**Table 4:** Logistic regression analysis for risk factors of anxiety.

**Variables**	**Univariate**		**Multivariate**	
	**OR (95% CI)**	***P*-value**	**OR (95% CI)**	***P*-value**
**Gender**				
Male	1			
Female	0.851 (0.656–1.120)	0.259		
**Grade**				
Freshmen	1.409 (0.973–2.038)	0.069		
Sophomores	1.083 (0.785–1.495)	0.627		
Juniors	1			
**Native place**				
Yunnan Province	1			
Outside Yunnan Province	1.096 (0.591 – 2.032)	0.772		
**Only child**				
Yes	0.820 (0.611–1.099)	0.184		
No	1			
**Smoking**				
None	1			
Once per week	0.467 (0.157–1.390)	0.171		
Twice per week	0.837 (0.252–2.784)	0.772		
More than twice per week	0.730 (0.372–1.435)	0.362		
**Drinking**				
None	1			
Once per week	1.193 (0.740–1.922)	0.469		
More than or equal to twice per week	1.002 (0.231–4.349)	0.997		
**Exercising**				
None	1.585 (0.981–2.561)	0.060	1.554 (0.970–2.489)	0.067
Once per week	0.726 (0.517–1.018)	0.064	0.720 (0.519–0.999)	0.049
Twice per week	0.710 (0.529–0.952)	0.022	0.706 (0.532–0.938)	0.016
More than twice per week	1		1	
**Taking medication**				
None	1		1	
Once per week	1.021 (0.756–1.380)	0.890	0.993 (0.742–1.329)	0.962
Twice per week	1.591 (0.967–2.619)	0.068	1.526 (0.940–2.479)	0.088
More than twice per week	2.519 (1.424–4.457)	0.002	2.310 (1.322–4.036)	0.003
**Irregular diet**				
None	1		1	
Once per week	1.205 (0.886–1.639)	0.235	1.164 (0.860–1.575)	0.326
Twice per week	1.329 (0.964–1.832)	0.083	1.320 (0.965–1.807)	0.083
More than twice per week	1.749 (1.148–2.664)	0.009	1.843 (1.225–2.773)	0.003
**Time spent on the smartphone**				
<30 min per day	1			
30–60 min per day	0.438 (0.159–1.207)	0.110		
1–2 h per day	0.640 (0.242–1.694)	0.369		
More than 2 h per day	0.651 (0.250–1.698)	0.380		
**Influence of COVID-19**				
None	1			
Small influence	1.304 (0.789–2.155)	0.300		
Moderate influence	1.565 (0.919–2.665)	0.100		
Great impact	1.785 (0.935–3.406)	0.079		
**Offline teaching**				
Well-adapted	1		1	
More adaptable	1.313 (0.875–1.971)	0.189	1.389 (0.939–2.055)	0.100
Almost inadaptable	1.897 (1.151–3.125)	0.012	2.225 (1.385–3.575)	0.001
Inadaptable	4.765 (1.553–14.617)	0.006	6.018 (1.990–18.203)	0.001
**Depression**				
No	1		1	
Yes	42.236 (27.238–65.493)	<0.001	42.480 (27.560–65.476)	<0.001

## Discussion

Our study showed that the prevalence of depression and anxiety was 52.5 and 29.6%, respectively. A wide variation in the prevalence of depression in University students ranges from 10 to 85% (17). The prevalence of depression and anxiety is in the range of a previous study for Chinese medical students (14), but the incidence is higher than that in our previous study (18). Inconsistent with a study in Pakistan (19), our results showed that the anxiety disorder prevalence is lower than that of depression. With the global burden of disease substantially growing (20), the prevalence of depression and anxiety is increasing year by year. It is meaningful for the sustainable development of student's mental health to analyze the causes and risk factors of the students' mental illnesses (21). Mapping the prevalence of depression and anxiety, like other serious diseases (22), in universities with incomplete registration can help guide more geographically precise public health intervention to support mental health-related care and reduce the prevalence.

Medical students who were freshmen and sophomores, exercised once per week, took medicine once per week, ate abnormally more than or equal to once per week, checked their smartphone more than 2 h per day, were concerned about the impact of COVID-19, considered offline teaching almost inadaptable, and were anxious had a higher risk of depression. Students who took medicine more than twice per week, ate abnormally more than twice per week, considered offline teaching inadaptable, and were depressive were more likely to have anxiety symptoms. Students who exercised once or twice per week had a lower risk of anxiety.

Our research has found that younger college students are more likely to be depressed. After entering college, the lifestyle and learning style of students change considerably (23). Because of the lack of parental care and teacher supervision on campus, college freshmen had to take time to adapt to their new living environment and the accompanying changes in learning styles; thus, first-year students face greater life stress and academic pressure (24). As learned helplessness theory supports, perceiving life stressors as uncontrollable results in depression for youth (25) when exposed to a new college campus environment. Freshmen or sophomores who encounter high levels of academic pressure without effective coping methods may experience depressive symptoms such as frustration, despair, and learning weariness (26). Additionally, the greater the academic stress they encounter, the higher the risk of depression-related problems they experience (27). A harmonious interpersonal relationship leads to a reduced likelihood of depression (28). Therefore, dealing with relationships between classmates and teachers is an enormous challenge for college students.

Physical exercise can play several positive roles, such as improving attention, executive function, and motor skills (29). The likelihood of depression, anxiety, and psychological disorder is higher for inactive individuals than for those who engage in a great deal of physical activity (30). Both acute exercise and long-term aerobic exercise are powerful forms to improve the brain-derived neurotrophic factor, a biomarker of depression in serum (31). In general, people with psychiatric disorders (including depression and anxiety) show lower levels of brain-derived neurotrophic factors in serum (32). This could be one reason why physical exercise improves the symptoms of depression.

Our data shows that students who took more pills were more anxious. Anxiety may come from disease and/or adverse drug reactions. Many diseases are accompanied by uncomfortable symptoms, such as fever, pain, and inflammation. Individuals with familial Mediterranean fever (33), chronic pain (34), and an increased inflammatory response (augmentation of interleukin-6, C-reactive proteins, and fibrinogen blood levels) (35) will experience anxiety symptoms at higher rates than the general population. In addition, dry eye disease may lead to emotional disorders such as anxiety due to the long-term symptoms of eye irritation (36). In addition to the anxiety caused by the disease itself, worrying about its long course and its impact on the body may also cause anxiety. Further, we need to consider adverse drug reactions when drugs are used to alleviate symptoms, for example, if the patient's depression and anxiety symptoms deteriorated after they took the second suvorexant dose or (more seriously) if this was accompanied by a new onset of suicidal thoughts (37). Another patient experienced poor judgment, psychomotor hyperactivity, and euphoria after 5 years of self-medication with prednisone and was subjected to depression and anxiety during temporary steroid withdrawal (38). Therefore, it is recommended that medical students seek medical treatment when they are sick and use drugs rationally under the guidance of doctors.

Irregular eating, anxiety, and depression are common psychiatric symptoms for people experiencing stress. Binge eating, the most unstable symptom, generally goes hand in hand with depression and anxiety (39). A negative self-image due to obesity caused by binge eating is a factor that leads to depression and anxiety. Body dissatisfaction in women with bulimia nervosa may lead to low self-esteem and indirectly result in depression (40). Eating disorders can change nucleotide metabolites and amino acid patterns in depression patients, with higher levels of asparagine, glutamine, proline, and arginine (41). Physical activity can improve the quality of life, symptoms of depression, and binge eating of adolescents with obesity (42), which is in line with our data about the relationship between physical exercise, irregular eating, and depression. Therefore, the right amount of regular physical exercise and diet is particularly vital to the mental health of medical students.

With the widespread use of smartphones, many people (especially college students) use phones for extended periods in daily life. For these students, the time for physical exercise and sleep is compressed during the COVID-19 pandemic (43). Increased screen time is associated with depression, anxiety, and stress scores among Mexican adults compared with decreasing leisure screen time (43). Additionally, decreasing physical activity is associated with higher stress scores overall. Physical activity and limited leisure screen time play a potential protective role on mental health, such as depression and anxiety (43), which is consistent with our study. For students, long screen time compresses sleep time, which causes a lack of sleep. Studies have reported that an increased risk of depressive symptoms was associated with poor sleep quality, including lack of sleep (44), although the mechanistic nature of such relationships remains unclear. The lack of sleep has been explored as a “secondary” factor to depression, and the risk of depression increases threefold in persons suffering from a lack of sleep (45). On the other hand, innovative technologies, including mobile phones, have led to the development of new industries and increased household income amid the COVID-19 crisis (46). Therefore, medical students need to keep a balance between the time spent on mobile phones, sleep quality, and physical exercise.

COVID-19 has had a significant impact on the way people live, sleep, exercise, and even eat. Additionally, the degree of concern about the negative impact of COVID-19 is a potential risk factor for lack of sleep and eating disorders. Lack of sleep coupled with eating disorders further increases the risk of anxiety and depression (47). An increase in the number of COVID-19 infections across the country has seriously affected the everyday lives of medical students at Kunming Medical University, although the number of infections in Yunnan is low. Teaching methods changed from traditional teaching in the classroom to teaching online. After some relief from the epidemic, teaching online switched back to teaching offline just as final exams were approaching. Many students could not adapt to this adjustment and faced great academic pressure. Academic stress is an influential factor causing anxiety and depression in college students. Moreover, anxiety can also positively and significantly predict depressive symptoms in the study (48), which is similar to our findings. Anxiety disorders are often comorbidities with depression, and in general, the onset of anxiety disorders precedes depression (49). Therefore, it is crucial to help medical students reduce the anxiety level caused by academic pressure and thus reduce the incidence of depression. The University could reduce academic pressure through academic counseling from teachers, an exchange of learning experiences among students, psychological counseling courses, and yoga meditation courses. Additionally, learning from the COVID-19 telephone-screening approach (50), telephone-based screening and triage can be used for students with mental illness, to provide a diagnosis or advice as soon as possible. Convenient and effective innovative technology measures should be strengthened to decrease the incidence of depression and anxiety and to improve the living condition of medical students.

### Limitations

This study has two limitations. First, the survey did not include all grade levels of medical students at Kunming Medical University. The respondents were medical students in their first, second, and third years. Senior undergraduate students were not included because it was difficult to collect data on senior students as they were scattered in various clinical hospitals. Our future research should be expanded to include medical students in all grades. Second, all the medical students were sampled from one medical University in Kunming, Yunnan Province. The research area should be expanded to include medical students in all medical universities in Yunnan, even in China. In our future studies, we will include subjects with a wider geographical range. Therefore, it is indeterminate whether our findings could be generalized to all medical students. However, our results still show the importance of monitoring and timely intervention in the mental health of medical students during the COVID-19 pandemic.

## Conclusions

In our study, depression and anxiety are more likely to be caused by low grades, lack of physical exercise, drug use, irregular diet, extensive screen time on mobile phones, being greatly affected by the COVID-19 pandemic, and inadaptability to offline courses. Additionally, depression and anxiety are highly comorbid. Our study identified a major mental health burden on medical students during the COVID-19 outbreak. More targeted measures should be taken to improve the mental state of the students to reduce the incidence of depression and anxiety. Because of the widespread influence of COVID-19, considerable efforts will be needed to accelerate progress.

## Data availability statement

The original contributions presented in the study are included in the article/supplementary material, further inquiries can be directed to the corresponding author/s.

## Ethics statement

The studies involving human participants were reviewed and approved by the Research Ethics Committees of Kunming Medical University (No.: KMMU2020MEC057). Written informed consent for participation was not required for this study in accordance with the national legislation and the institutional requirements.

## Author contributions

YG, SL, LZ, JX, HS, and YY were involved in setting up the study and data collection. QX, LH, QY, JM, LP, YX, JY, and HY contributed to the data analysis. YG drafted the manuscript. All authors commented on subsequent versions, contributed to the article, and approved the submitted version.

## Funding

This work was supported by National Natural Science Foundation of China (82060650), Applied Basic Research Project of Yunnan (202001AT070137), Development and Application of Biotic Resource in Yunnan Province (202002AA100007), Yunnan Key Laboratory of Pharmacology for Natural Products Fund Project, and Program Innovative Research Team in Science and Technology in Yunnan Province (202005AE160004).

## Conflict of interest

The authors declare that the research was conducted in the absence of any commercial or financial relationships that could be construed as a potential conflict of interest.

## Publisher's note

All claims expressed in this article are solely those of the authors and do not necessarily represent those of their affiliated organizations, or those of the publisher, the editors and the reviewers. Any product that may be evaluated in this article, or claim that may be made by its manufacturer, is not guaranteed or endorsed by the publisher.
